# Visual Working Memory Load-Related Changes in Neural Activity and Functional Connectivity

**DOI:** 10.1371/journal.pone.0022357

**Published:** 2011-07-18

**Authors:** Ling Li, Jin-Xiang Zhang, Tao Jiang

**Affiliations:** Key Laboratory for NeuroInformation of Ministry of Education, School of Life Science and Technology, University of Electronic Science and Technology of China, Chengdu, China; Beijing Normal University, China

## Abstract

**Background:**

Visual working memory (VWM) helps us store visual information to prepare for subsequent behavior. The neuronal mechanisms for sustaining coherent visual information and the mechanisms for limited VWM capacity have remained uncharacterized. Although numerous studies have utilized behavioral accuracy, neural activity, and connectivity to explore the mechanism of VWM retention, little is known about the load-related changes in functional connectivity for hemi-field VWM retention.

**Methodology/Principal Findings:**

In this study, we recorded electroencephalography (EEG) from 14 normal young adults while they performed a bilateral visual field memory task. Subjects had more rapid and accurate responses to the left visual field (LVF) memory condition. The difference in mean amplitude between the ipsilateral and contralateral event-related potential (ERP) at parietal-occipital electrodes in retention interval period was obtained with six different memory loads. Functional connectivity between 128 scalp regions was measured by EEG phase synchronization in the theta- (4–8 Hz), alpha- (8–12 Hz), beta- (12–32 Hz), and gamma- (32–40 Hz) frequency bands. The resulting matrices were converted to graphs, and mean degree, clustering coefficient and shortest path length was computed as a function of memory load. The results showed that brain networks of theta-, alpha-, beta-, and gamma- frequency bands were load-dependent and visual-field dependent. The networks of theta- and alpha- bands phase synchrony were most predominant in retention period for right visual field (RVF) WM than for LVF WM. Furthermore, only for RVF memory condition, brain network density of theta-band during the retention interval were linked to the delay of behavior reaction time, and the topological property of alpha-band network was negative correlation with behavior accuracy.

**Conclusions/Significance:**

We suggest that the differences in theta- and alpha- bands between LVF and RVF conditions in functional connectivity and topological properties during retention period may result in the decline of behavioral performance in RVF task.

## Introduction

Visual working memory (VWM) allows for temporary storage and manipulation of the visual information necessary for the subsequent complex cognitive tasks [1]. The neuronal mechanisms required to sustain and process coherent visual information and the mechanisms of limited VWM capacity have remained unknown. Studies have reported that the capacity of VWM is limited to a fixed number of objects, up to about 4 objects, and varies by object complexity [2]–[3]. Although numerous studies have utilized behavioral accuracy by Cowan's formula to estimate VWM capacity, using neural activity during the retention interval to predict individual memory capacity was proposed recently [4]–[5]. During the retention interval, neural activity was strongly modulated by the memory load, and was invariable when the memory load reached the memory capacity [4].

Behavioral studies have found that the increasing memory load results in the decline of accuracy and the increase of reaction time. Neuroimaging studies (including functional magnetic resonance imaging, fMRI; electroencephalography, EEG; magnetoencephalography, MEG) have shown that a network of several cortical regions in the frontal, temporal, parietal and occipital lobes supports the neural activity of VWM system [5]–[8], in which the prefrontal, parietal, and occipital areas play an important role in encoding and maintenance of objects in visual memory [4], [6], [9]. Because of the low temporal resolution for fMRI technology, it is difficult to reveal the neural mechanism that occurs instantaneously during the retention interval. Hence, EEG/MEG or simultaneous MEG and EEG technology are selected to record the neural activity when subjects are performing a VWM task. Due to the low spatial resolution for EEG/MEG, multi-channel measurement and the technology of minimizing the contribution of volume conduction should be considered and applied. Prior studies have shown that, during retention interval in a short-term memory task in humans, posterior alpha (9–12 Hz) and frontal theta (4–7 Hz) power systematically increase with the increase in working memory load [10]–[11], and enhanced gamma (20–80 Hz) activity at frontal and left posterior sites appears in the memory condition [12]. However, little is known about how the VWM network of those functionally distinct areas operates during the retention interval for hemi-field WM. Graph theoretical method provides a powerful novel way of quantifying the structural and functional brain networks using structural MRI, diffusion MRI, functional MRI and EEG/MEG techniques [13]–[16].

EEG/MEG studies based on graph theoretical method have been increasingly performed to investigate brain functional connectivity with the advantage of high temporal resolution and broad frequency band [17]–[18]. For EEG data, each recorded electrode represents a vertex of a graph, and an edge of a graph indicates strong functional correlativity between two channels. Functional connectivity between all pairs of electrodes has been estimated using different measurements, including linear temporal correlation, generalized synchronization, fluctuation analysis, and phase synchronization [19]–[22]. The resulting connectivity matrixes are converted to graphs by thresholding. The graphs are then characterized by degree, clustering coefficient, and shortest path length, among other measures. Because oscillatory phase synchrony is thought to be very valuable for the investigation of neuronal interactions and communications [23], in the present study, we used this method to measure functional connectivity. An EEG study has reported a gamma synchronous network oriented to the cued location using phase synchronization [24], but it did not quantify this large-scale network by graph theoretical methods. A study of simultaneous MEG and EEG technology investigated the structure of oscillatory phase synchronized networks during the VWM retention interval, and found that alpha- (7–13 Hz) and beta- (16–25 Hz) bands networks had a memory-load dependent, scale-free small-world structure [25]. In addition, one other study used a similar method indexing connection density and found that human cortical alpha- (10–13 Hz), beta- (18–24 Hz), and gamma- (30–40 Hz) bands among fronto-parietal and visual areas were memory-load dependent during the VWM retention interval [5]. However, the above-mentioned studies used topological parameters of a graph to characterize the system level mechanisms of VWM maintenance based on a delayed matching-to-sample task in the whole visual-field memory task.

For ERP studies of the whole visual-field WM, a broadly distributed sustained negative wave has been reported to be modulated by the memory load during the memory retention period [26]–[27]. For ERP studies of the hemi-field VWM, a contralateral delay activity (CDA) across the posterior parietal, posterior temporal and occipital electrode sites, or sustained posterior contralateral negativity (SPCN), were found in memory retention period [4], [28]–[29]. And they found that the CDA amplitude was modulated by the number of memory items and sensitivity to individual differences in memory capacity. In the present study, we performed a hemi-field VWM task, and addressed the following hypotheses: 1) VWM load-related changes during the retention interval results in an alteration of contralateral delay activity (CDA) at parietal-occipital areas, which may be used to predict mean memory capacity; 2) the functional connectivity of whole brain networks for the hemi-field VWM during the retention interval is VWM load-dependent, and modulated in different ways for different frequency bands; 3) the changes in brain functional networks are dependent on the direction of visual-field WM during the retention interval with increasing memory load. To test these hypotheses, neural activity was measured by ERPs at parietal-occipital areas, and functional connectivity was estimated by calculating the phase synchrony between each pairwise combination of 128 electrodes sites. The resulting connectivity matrices were converted to a set of undirected binary graphs by set thresholds. Following that, the mean degree, clustering coefficient, and the shortest path length of a graph were evaluated.

## Materials and Methods

### Subjects

Fourteen right-handed subjects (three females) participated in the present study for monetary compensation. They were recruited by advertisement at the University of Electronic Science and Technology of China. The mean age was 23 years, ranged between 21 and 28 years. An informed consent was signed by each subject before the experiment. The subjects had no history of neurological problems and had normal color vision. The local committee for the protection of human subjects for the University of Electronic Science and Technology of China approved the study.

### Experimental paradigm

**Figure 1:**
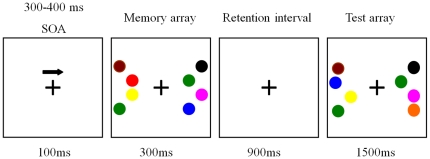
Experimental paradigm. Each trial began with a cue, and then the memory array (300 ms) was presented. Following that, there was a 900-ms retention interval and then the presentation of the test array.

Figure 1 illustrates an example of the stimulus sequence. Each trial began with a black fixation cross and arrow (100 ms) instructing subjects to attend and remember the items in the corresponding visual field. Following that, the memory array (300 ms) was presented. There was the same number of circles for the left and right visual field, ranging from 1 to 6 circles with different colors. There were 9 colors in total, including black, pink, red, orange, yellow, green, blue, cyan, and carmine. The diameter of each circle was 2.2 cm. The circles were presented randomly on the locations of an invisible 4×3 matrix (5.5°×4.2°) in the left and right visual field. There was a 900 ms retention interval and then the presentation of the test array. The test array remained on the screen for up to 1500 ms or until subjects responded with a button press, indicating whether the colors of circles in attended hemi-field were the same or different from those in the memory array. There were four equiprobable conditions: the same colors and locations were present in both the test and the memory array in attended hemi-field, only the color of one circle was changed in the test compared with the memory array in attended hemi-field, only the location of one circle was changed in the test compared with the memory array in attended hemi-field, and both color and location of one circle was changed in the test compared with the memory array in attended hemi-field. For unattended hemi-field, there were the similar four conditions as attended hemi-field.

During the experiment, subjects were told to remember color and disregard location. Subjects used their right hand to press button 1 when the color of all circles in the memory array was the same as that in the test array in the attended visual field and button 2 when a color was changed. Subjects were required to maintain central fixation throughout the recordings and to respond as quickly as possible. Two practice blocks were done before starting the experimental trials to learn the task. There were 24 experimental blocks for each subject, each block consisting of 60 trials. Each subject performed 1440 trials in total, with memory load ranging from 1 to 6 items, in either the left or right visual field, so that each condition had 120 trials. Stimuli were presented and behavioral results were recorded and analyzed using E-prime software.

### Data collection and preprocessing

EEG was recorded using the EGI system with a 128 channel electrode cap. The reference electrode was the Cz (129^th^) electrode, and EOG were recorded simultaneously from electrodes placed above and below the left eye. All electrode impedances were kept well below 15 kΩ. EEG was digitized at 1000 Hz with an amplifier band-pass of 0.1–48 Hz, segmented from 200 ms before the onset of the memory array to 1500 ms after the memory array onset. EOG and significant muscle artifact were excluded by automatic artifact rejection (±100 µV). EEG epochs containing incorrect button press were excluded. The data was baseline corrected using the 200 ms before the onset of the memory array. After this preprocessing, 1611, 1579, 1414, 1241, 1132, and 1036 for left visual field trials remained in total over all subjects for memory loads of 1 to 6, respectively. For the right visual field, 1596, 1564, 1378, 1193, 1122, and 1003 trials remained in total over all subjects for memory loads of 1 to 6, respectively. Those EEG data were used for the further ERP and phase synchronization analysis. For each subject, at least 64 trials were included for each condition.

### Behavioral data analysis

Mean reaction times (RT) and accuracy were calculated for 6 memory loads in LVF and RVF memory tasks across all subjects. RT and accuracy were assessed by repeated measure analysis of variance (ANOVA) for memory loads 1 to 6 in both visual-field conditions. Kendall's coefficient of concordance (*W*) test was applied to measure the relationships across the six memory load conditions with RT and accuracy. The capacity of visual working memory was estimated using a formula defined by Cowan [30].

### ERP analysis

Single trials from each subject in the 1 to 6 memory load conditions in the left and right visual-field conditions were averaged, respectively. Considering the primarily contralateral organization of the visual system responses to contralateral and ipsilateral memory arrays could be measured by the corresponding electrode position. For each memory load, contralateral and ipsilateral ERP waveforms were calculated and analyzed at lateral parietal-occipital electrodes (PO7 and PO8). The contralateral ERP was the mean of ERP at PO7 for right visual field memory arrays and the mean ERP at PO8 for left visual field memory arrays. In contrast, the ipsilateral ERP was the mean ERP at PO8 for right visual field memory arrays and the mean ERP at PO7 for left visual field memory arrays. The difference waves between ipsilateral and contralateral ERP waveforms were analyzed for each memory load. In order to compare the amplitude of the ERP difference waves during the memory retention interval for the 6 memory load conditions, the mean amplitude was calculated within two measurement windows of 300–800 ms and 800–1200 ms after the onset of the memory array. Mean amplitudes were assessed by repeated measure analysis of variance (ANOVA), Kendall's coefficient of concordance (*W*) test and Pearson correlation among six memory load conditions.

### Computation of scalp current density

To minimize the contribution of volume conduction and remove spurious synchronization, the following steps were applied to single trial of EEG data before the computation of the phase synchrony. Step 1. Each single trial of 12 conditions (6×2, memory load and visual field) of every subject was filtered by the band-pass finite impulse response filters at 4 Hz intervals between 4 and 40 Hz. Totally signals of 9 frequency bands were obtained. Step 2. To minimize the modulated effect to phase by the relative amplitudes of sources, we standardized the EEG amplitudes. This was done by subtracting the mean amplitude at a given frequency band in the cue interval (−100 ms ∼0 ms) from the amplitude and dividing the standard deviation of amplitude at that frequency in the cue interval for every time point. Step 3. We used a current source density (CSD) toolbox of MATLAB supplied by Kayser J. (http://psychophysiology.cpmc.columbia.edu/Software/CSDtoolbox/index.html) that implements a spherical spline algorithm of Perrin et al. to estimate scalp current density (SCD) for EEG data [31]–[32]. The spline interpolation constant was set to 4.

### Computation of phase synchrony

After above SCD computation, the data from −100 ms before the onset of the memory array to 1200 ms after the onset of the memory array were used to estimate long-range neural phase synchrony by calculating mean resultant length (also called phase-locking value). Under different names, the mean resultant length (MRL) has been used to measure the bivariate phase synchronization in a number of EEG studies [33]–[36]. MRL was obtained by comparing the instantaneous phases of pairs of signals. MRL between electrodes *j* and *k*, at each sample time *t*, across the *N* trials, were quantified as [33]

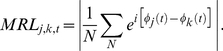
(1)


Instantaneous phase *ø*(*t*) of a signal *x*(t) was estimated by Hilbert transform H 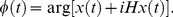
(2)


MRL takes on values between 0 and 1, describing a continuum between no and perfect phase synchronization. Strong synchronization means a concentrated distribution on one side, and weak synchronization corresponds to a nearly uniform distribution of the phase difference on the unit circle.

For baseline compensation, we used the cue interval (−100 ms ∼0 ms) as the baseline. Baseline corrected was done by subtracting the mean MRL at a given frequency band in the cue interval from the MRL and dividing the standard deviation of MRL at that frequency in the cue interval for every time point.

To compensate for the phase synchrony caused by stimulus stimulus-evoked activity, we created 200 shuffling SCD data for each frequency and time point. 200 MRL values were calculated using above method, and the surrogate distributions of MRL values were obtained [33]. Negative MRL values which mean decrease in phase synchronization were not discussed in present study, so that those values were set to zeros. For positive MRL values, we used 95th percentile of the surrogate distribution as the synchronous criterion. MRL values that met or exceeded this criterion were discussed and kept, and other MRL values were set to zeros. Finally, significant MRL values were normalized by dividing the maximum value of MRL in all conditions.

The result of computing the normalized significantly corrected MRL for all pair-wise combinations of channels is a square 128×128 matrix for each of 1200 time points (0 ms ∼1200 ms). After above processing, therefore 1512 temporal phase synchronization matrices (128×128×1200) for 12 conditions (6 memory load conditions and 2 visual fields) in 14 subjects in 9 frequency bands were obtained.

### Computation of the mean degree *K*, clustering coefficient *C*, and shortest path length *L* of a graph

A phase synchronization matrix can be converted to a binary graph by defining vertices (electrodes) and edges. If a value of phase synchronization between electrodes *i* and *j* exceeds values of threshold *T*, an edge exists between *i* and *j* vertex. These vertexes and edges construct a functional brain network. However there is no unique way to choose *T*.

After obtaining a graph from the synchronization matrix, the mean degree *K*, clustering coefficient *C* and shortest path length *L* were measured to characterize the functional connectivity of the human brain [14]–[16]. The degree at each vertex, *k_i_*, *i* = 1, 2… *M* (*M* = 128), is defined as the number of other vertexes connecting with the vertex *i*. The degree of a graph, *K*, is the average of the degrees of all the vertexes in the graph



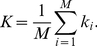
(3)


For vertex *i*, suppose that the degree *k_i_*, indicates the maximum possible number of edges between these *k_i_* vertexes is *k_i_*(*k_i_*−1)/2. In fact, the number of existing edges between neighbours of vertex *i* is *E_i_* [37]–[38]. The clustering coefficient *C_i_* of a vertex *i* with degree *k_i_* is defined as 
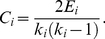
(4)


Hence, clustering coefficient *C* of a graph is averaged over all vertices of the graph.



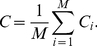
(5)


The path length *d_ij_* between two vertices *i* and *j* is the minimal number of edges that have to be traveled to go from *i* to *j*. Shortest path length *L* of a graph is the mean of the path lengths between all possible pairs of vertices:



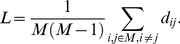
(6)


The degree, clustering coefficient and path length are the core measures of graphs. Higher *K* reflects more connections in a graph. *C* is an index of local structure and a measurement of resilience to random error. In contrast, *L* is a global characteristic and indicates the routing efficiency of the network.

### Direct comparisons of the mean degree *K* for the phase synchrony data

We analyzed the phase synchrony data at the graph level by using the mean degree *K*, to identify the relationships between connections from all possible pairwise interactions among 128 brain areas and three factors of visual field, memory load, and frequency bands. Corrected MRL matrices had been tested and the positive value in MRL matrices indicated significant phase synchronization between two brain areas. Hence, an edge exists between *i* and *j* vertex when the MRL value is bigger than zero. Therefore 1512 temporal graphs (128×128, 500 time points (300∼800 ms)) for 12 conditions (6 memory load conditions and 2 visual fields) in 14 subjects of 9 frequency bands were constructed. Then 1512 mean degree *K* was calculated for each time point and then averaged over 500 time points. Kendall's coefficient of concordance (*W*) test and Pearson correlation were performed to identify the correlations between *K* and three factors (two visual fields, nine frequency bands, and six memory loads). In order to obtain the neural correlates with behavioral response, multi-linear regressions of the *K* and memory load, with the behavioral results (RT and accuracy) were performed in all significant frequency bands, respectively. As the possible 15 correlations among 6 memory loads (6×5/2 = 15) were subjected to multiple, non-independent tests, we employed the Bonferroni correction for multiple comparisons (i.e., 0.05/15 = 0.0033 as threshold).

### Graph visualization

Matlab software was used for graph visualization for averaged graphs. The region of each vertex was positioned according to its electrode location in the two-dimensional coordinates. The Cartesian coordinate origin was the midpoint between the left and right earlobes. The axis that was directed away from the origin towards the nasion was the +x axis, and the axis that was directed away from the origin towards the left earlobe was the +y axis. Hubs in averaged graph were found as vertexes whose degrees were larger than the average degree of the graph.

### Comparisons of the *C* and *L* in the same mean degree *K*

Considering the strong influence of the connectivity density on the topological properties of the brain functional networks [17]–[18], [39], clustering coefficient *C*, and path length *L* of 1512 phase synchronization graphs in retention period (6 memory load conditions, 2 visual fields, 14 subjects, 9 frequency bands, at 300∼800 ms time points) were calculated and compared when the mean degree *K* in each memory load were the same (minimum mean degree for the phase synchrony data in six loads). We applied different values of threshold *T* to each graph to obtain the same mean *K*. Because we focused on the changes of *C*, and *L* in retention period, then, we averaged the *C*, and *L* across 500 time points. The mean *L*, and *C*, among six memory loads, two visual fields, and 9 frequency bands, were assessed by Kendall's coefficient of concordance (*W*) and Pearson correlation, which tested whether those three factors had significantly different influence on both topological parameters, respectively. Multi-linear regressions of the *C* (or *L*) and memory load with the behavioral results (RT and accuracy) were also performed in all significant frequency bands, respectively. We employed the Bonferroni correction for multiple comparisons (i.e., 0.05/15 = 0.0033 as threshold), too.

### Identifying small-world brain networks

A small world network has a *C* close to that of an ordered network, but a very small *L* close to that of a random network. In order to identify small-world brain networks from ordered and random networks, we calculated the values of clustering coefficient *C* and path length *L* in both types of network. Theoretical ordered networks have a high *C_ordered_* (0.75) and a large *L_ordered_* (*M*/2*K*) [37]. In a theoretical random network, *C_random_* (*K*/*M*) is very small and *L_random_* (In(*M*)/In(*K*)) is very short [37]. However, the theoretical networks have Gaussian degree distributions that may differ from the degree distribution of actual brain networks. We have obtained the *C_exp_* and *L_exp_* of brain networks for each condition of each mean degree *K* in the above section. Hence, we generated 500 random and ordered control networks for corresponding networks by a Markov-chain algorithm which preserves the degree distribution exactly [40]. And the mean *C_exp_*, *L_exp_ C_ordered_*, *L_ordered_*, *C_random_*, and *L_random_* across six memory loads for nine frequency bands and two visual-field conditions in all subjects were calculated. Paired *t*-test was used to identify the small-world-ness in different conditions. As the possible 15 correlations among 6 memory loads (6×5/2 = 15) were subjected to multiple, non-independent tests for the mean *C* or *L*, we employed the Bonferroni correction for multiple comparisons (i.e., 0.05/15 = 0.0033 as threshold).

## Results

### Behavioral results

Mean reaction time (RT) in the VWM task ascended with increasing memory load. Mean RT was 529.20±30.37 ms (mean ± standard error of the mean (SEM) for this and all following results), 593.04±33.60 ms, 660.40±38.78 ms, 691.99±39.88 ms, 714.74±40.97 ms, and 730.29±44.20 ms from the memory load 1 to 6 across both visual-field conditions, respectively. Correspondingly, mean accuracy declined with increasing memory load. Mean accuracy was 95.63±0.69%, 93.54±0.68%, 83.13±0.77%, 72.47±1.09%, 67.11±1.22%, and 60.68±0.86% from the memory load 1 to 6, respectively.

**Figure 2:**
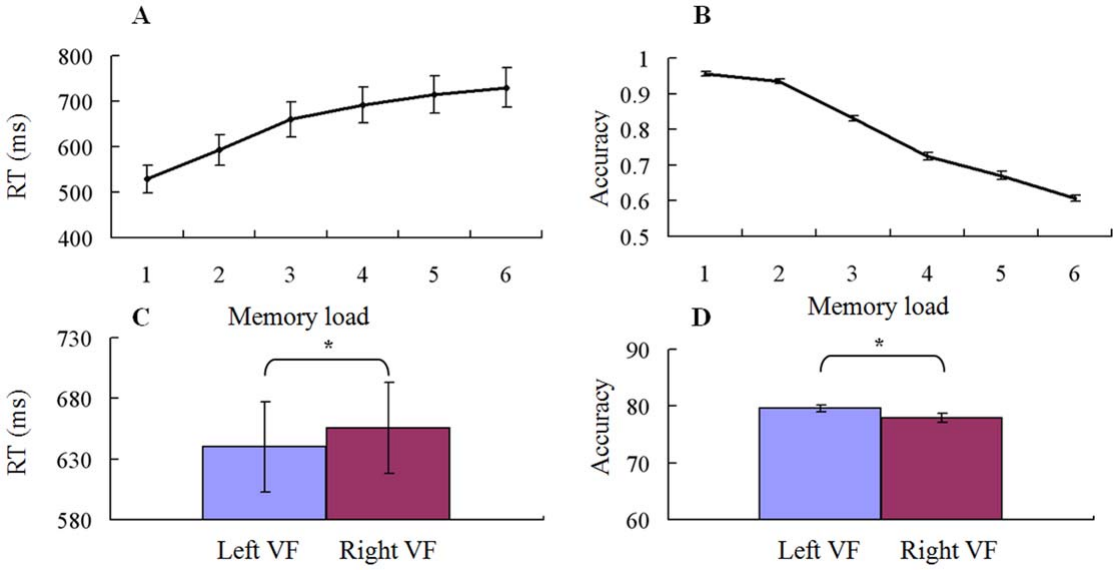
Behavioral responses. (A) Average reaction time (RT) and (B) accuracy are shown for the 14 subjects in six memory load conditions across both visual-field conditions. Subjects responded more quickly and accurately in the lower memory load. (C) Average RT and (D) accuracy are shown for all subjects in left and right visual filed conditions across six load conditions (* p<0.01). Subjects responded more quickly and accurately in the left visual field condition. Error bars shows SEM of within-subject.

Figure 2A–B shows the RT and accuracy for memory loads from 1 to 6 over two visual-field conditions. Mean RTs increased significantly with increasing memory load (a repeated-measure ANOVA: all paired memory load conditions, p<0.05; Kendall's coefficient of concordance test: *W* = 0.897, p<0.001). Mean accuracy decreased significantly with increasing memory load (ANOVA: p<0.05; Kendall's *W* = 0.977, p<0.001). Cowan's value increased significantly from load 1 to 2, load 2 to 3 (ANOVA: p<0.001), and reached a plateau at load 3 (p>0.05 for the differences between load 3 and 4 condition). Cowan's value in memory load 3 was about 2. Cowan's value significantly depended on the memory load conditions (Kendall's *W* = 0.683, p<0.001). The subjects' mean memory capacity was 1.98±0.05.

Figure 2C–D shows the average RT and accuracy for all memory loads in LVF and RVF conditions. Mean RT was 640.00±36.95 ms and 655.78±37.59 ms, and mean accuracy was 79.58±0.62% and 77.94±0.77%. Subjects responded more quickly and accurately in the LVF memory condition than the RVF condition (Paired *t* test: *t*_(13)_ = 3.92, p = 0.0017 for RT; *t*_(13)_ = 3.14, p = 0.0078 for accuracy).

### ERP results

Figure S1 shows the grand averaged contralateral and ipsilateral ERP waveforms for the memory load of 3 condition at the lateral occipital and parietal electrodes (PO7 and PO8). During the period of retention interval, a larger negative-going voltage was found in contralateral ERP compared to ipsilateral ERP. Figure S2 shows the ERP difference waves at PO7/8 electrodes for memory load 1, 2, 3, and 5 conditions. Approximately 300 ms after the onset of the memory array, the amplitude of ERP difference was highly sensitive to the number of items. Increasing memory load from 1 to 2 or from 2 to 3 resulted in an increase in absolute amplitude.

**Figure 3:**
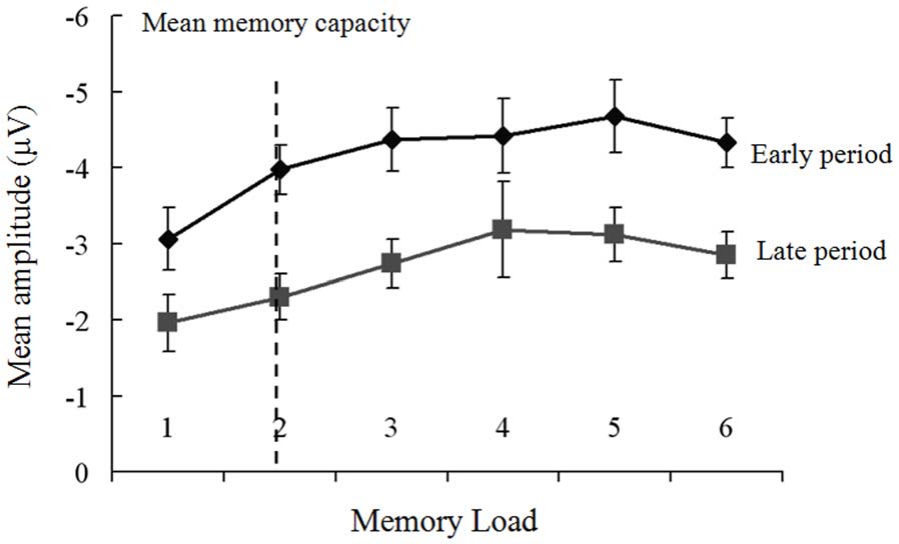
Mean amplitude of ERP difference. The mean difference between the ipsilateral and contralateral waveforms within the windows of 300–800 ms (early) and 800–1200 ms (late) after the onset of the memory array as a function of memory load. Mean amplitude increased with increasing memory load, and reached a plateau at load 2 condition for both periods.

We did analysis with different time-windows on ERP difference waves (early period: 300∼800 ms, and late period: 800∼1200 ms after the onset of the memory array). Figure 3 illustrates the mean amplitude of ERP difference in the early and late time-windows. Mean amplitude was −3.06±0.41 µV, −3.97±0.33 µV, −4.37±0.42 µV, −4.42±0.50 µV, −4.68±0.48 µV, and −4.33±0.33 µV from memory load 1 to 6 for early period, and −1.96±0.37 µV, −2.30±0.30 µV, −2.74±0.31 µV, −3.18±0.63 µV, −3.12±0.35 µV, and −2.84±0.31 µV for late period, respectively. During the late period mean amplitude of difference waves decreased significantly compared with that in the early period (Paired *t* test: p<0.001). These results showed that memory retention initially began as contralateral predominance, but tended to decrease after a period of time.

For the early period, there were significant differences between memory loads 1 and 2, 3, 4, 5, 6 conditions (ANOVA: p<0.01), respectively, but there was no difference among load 2, 3, 4, 5, 6 conditions. Mean amplitude depended significantly on the memory load (Kendall's *W* = 0.412, p<0.0001, six memory load conditions). However, Kendall's *W* was very low when we only tested conditions of memory load from 2 to 6 (*W* = 0.120, p = 0.150). For the late period, there were significant differences between memory loads 1 and 3, 4, 5, 6 conditions (ANOVA: p<0.01), respectively, but there was no significant difference between memory load 1 and 2 (p = 0.25). Mean amplitude depended significantly on the memory load (Kendall's *W* = 0.229, p = 0.007, six memory load conditions). However, Kendall's *W* was very low when we only tested conditions of memory load from 2 to 6 (*W* = 0.099, p = 0.236). We used Pearson correlation to test the relationship between absolute value of difference waves with memory load. A significantly positive correlation was found for the early (two-tailed, p = 0.016) and late (p = 0.029) periods. Hence, during the early or late period, mean absolute amplitude increased with the increase of memory load and reached a plateau at the condition of memory load 2, which was close to the mean memory capacity of present subjects.

### The results of the mean degree *K* for the phase synchrony data

**Figure 4:**
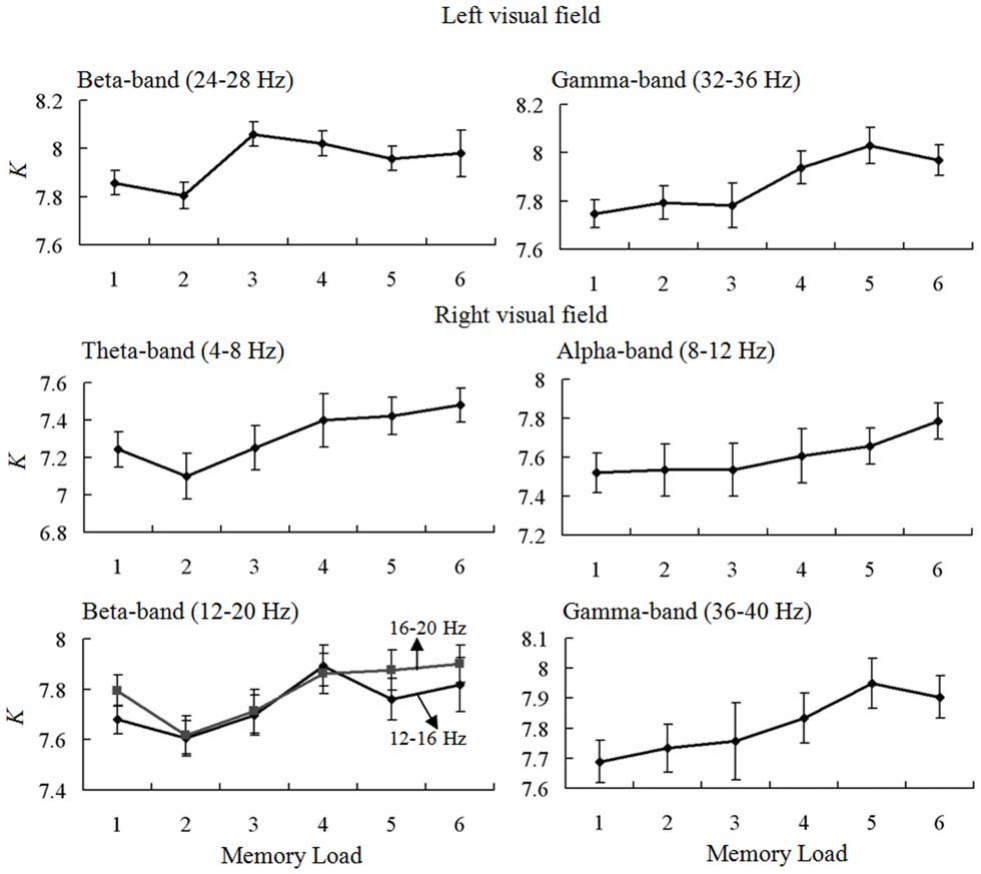
Mean degree *K* of a brain functional network. Mean degree *K* as a function of memory load during the retention period for left visual field memory condition (*first row*), and right visual field condition (*second and third rows*) in memory load-dependent frequency bands. The results of Kendall's coefficient of concordance (*W*) test and Pearson correlation indicated that increasing memory load increased mean degree *K* in those frequency bands.

We obtained 1512 mean degree *K* under the condition of two visual fields, nine frequency bands, six memory loads, and 14 subjects. For the LVF condition, Kendall's *W* of mean degree *K* showed that *K* was memory load-dependent in beta- (24–28 Hz) and gamma- (32–36 Hz) frequency bands (for beta-band, *W* = 0.167, p = 0.039; for gamma-band, *W* = 0.244, p = 0.004). For the RVF condition, Kendall's *W* of mean degree *K* showed that *K* was memory load-dependent in theta- (4–8 Hz), alpha- (8–12 Hz), beta- (12–20 Hz), and gamma- (36–40 Hz) frequency bands (for theta-band, *W* = 0.228, p = 0.007; for alpha-band, *W* = 0.155, p = 0.05; for low beta-band (12–16 Hz), *W* = 0.174, p = 0.033; for high beta-band (16–20 Hz), *W* = 0.160, p = 0.048; for gamma-band, *W* = 0.159, p = 0.049). Figure 4 illustrates the mean degree *K* as a function of memory load for significant frequency bands. Significantly positive Pearson correlation coefficients were found for those frequency bands (p<0.05). The results of Kendall's coefficient of concordance (*W*) test and Pearson correlation indicated that increasing memory load increased mean degree *K* in beta- and gamma- frequency bands for LVF memory condition, but theta-, alpha-, beta-, and gamma- frequency bands for RVF memory condition.

**Figure 5:**
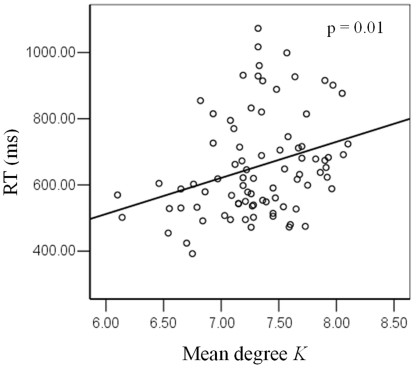
Relationship between the mean reaction time and mean degree *K*. Linear regression between mean reaction time (RT) and mean degree *K* in theta-band for right visual field condition across six memory load conditions. A linear regression analysis was conducted to compare the relationship between RT and *K*. Each single subject in each memory load condition is marked as a circle, totaling 84 circles. This regression was significant (p = 0.01, Bonferroni corrected), indicating a positive linear correlation between RT and *K*.

Multi-linear regressions of the mean degree *K* and memory load with the behavioral results (RT and accuracy) were performed in all significant frequency bands, respectively. Only in the theta-band and for the RVF memory condition there was a significant multi-linear regression between RT and two independents (*K* and memory load) (p<0.0001). Partial regression coefficients of mean degree *K* (p = 0.04) and memory load (p = 0.001) were significant too. This linear regression was significant between RT and *K* (p = 0.01, Bonferroni corrected). Figure 5 illustrates the linear relationship between mean degree *K* and RT for theta-band in RVF condition. Those results indicated that reaction time was not only dependent on the memory load but also the mean degree *K* of the brain functional networks. Increasing mean degree *K* increased reaction time in theta-band for RVF memory condition.

### The results of mean graph visualization

The mean brain functional network was calculated by averaging the phase synchrony graphs across all the subjects, six memory loads, and 500 time points in retention period (300∼800 ms) within two visual-field conditions for four frequency bands (theta, alpha, beta, and gamma). We then used 0.07 as a threshold to all mean networks to create the graphs. Figure S3 shows the two-dimension map of scalp electrode locations and the regions of interest (ROI). Figure S4 illustrates the connective networks for four frequency bands in left and right visual-field conditions under the same threshold condition. The networks showed different connective density for two visual-field memory conditions in each frequency bands. The networks of LVF memory had larger connective density than those of RVF condition. Alpha-band network had maximal phase synchronization, following theta-, gamma-, and beta- bands. Furthermore, in Figure S4, beta-band network was obviously interhemispheric connections. LVF memory retention evoked more phase synchronization between right posterior and left frontal brain regions, and RVF memory retention evoked more phase synchronization between left posterior and right frontal brain regions.

Then, to quantify the significance of brain regions in functional connectivity, 34 scalp electrodes were selected to categorize into eight main brain regions (see Figure S3). Table 1 illustrates the region locations with averaged degree *K* in selected electrodes. Specially, we defined a hub as a vertex whose degree is larger than the mean degree of the network. For theta-band, bilateral frontal regions were hubs in both hemi-field conditions, but bilateral occipital regions were hubs only in RVF condition. For alpha-band, bilateral frontal, occipital regions had high degree in both hemi-field conditions, but only left temporal regions of LVF and only left parietal regions of RVF were hubs. For beta-band, bilateral occipital and memorial ipsilateral frontal areas were hubs in both hemi-field conditions, but only right temporal regions of LVF and only left parietal regions of RVF were hubs. For gamma-band, right occipital and parietal areas were hubs in both hemi-field conditions, but right frontal and left parietal regions of RVF were hubs too. From Table 1, we found that bilateral frontal and occipital areas were very important for both hemi-fields memory retention, and left parietal areas especially played a key role in RVF memory retention.

**Table 1:** Summary of network measures for each condition.

Region name	Degree (*K*)							
	Left visual field				Right visual field			
	Theta	Alpha	Beta	Gamma	Theta	Alpha	Beta	Gamma
Left frontal	***10.50***	***17.83***	***2.50***	3.33	***7.17***	***13.67***	1.00	1.50
Left parietal	3.50	6.25	1.00	2.50	3.50	***11.50***	***3.25***	***4.50***
Left temporal	4.00	***13.33***	1.67	3.00	4.00	10.00	0.67	3.67
Left occipital	5.00	***16.75***	***2.50***	3.75	***5.00***	***17.75***	***2.25***	3.25
Right frontal	***9.33***	***13.33***	1.00	3.83	***6.67***	***12.67***	***2.00***	***6.17***
Right parietal	1.50	9.50	1.00	***5.50***	2.75	8.00	0.75	***5.75***
Right temporal	3.33	10.33	***2.00***	1.00	1.33	8.67	0.67	3.33
Right occipital	5.50	***13.00***	***3.50***	***6.00***	***4.75***	***19.00***	***1.50***	4.00

Averaged degree *K* in eight brain regions of selected electrodes was shown and network hubs were listed in bold and italic.

### The results of *C* and *L* under the same mean degree *K*

1512 mean clustering coefficient (*C)* and shortest path length (*L)* measurements during the retention period (300∼800 ms) were obtained from the phase synchrony matrix under the same mean degree *K* for each subject, each visual-field, each memory load condition, and each frequency band.

**Figure 6:**
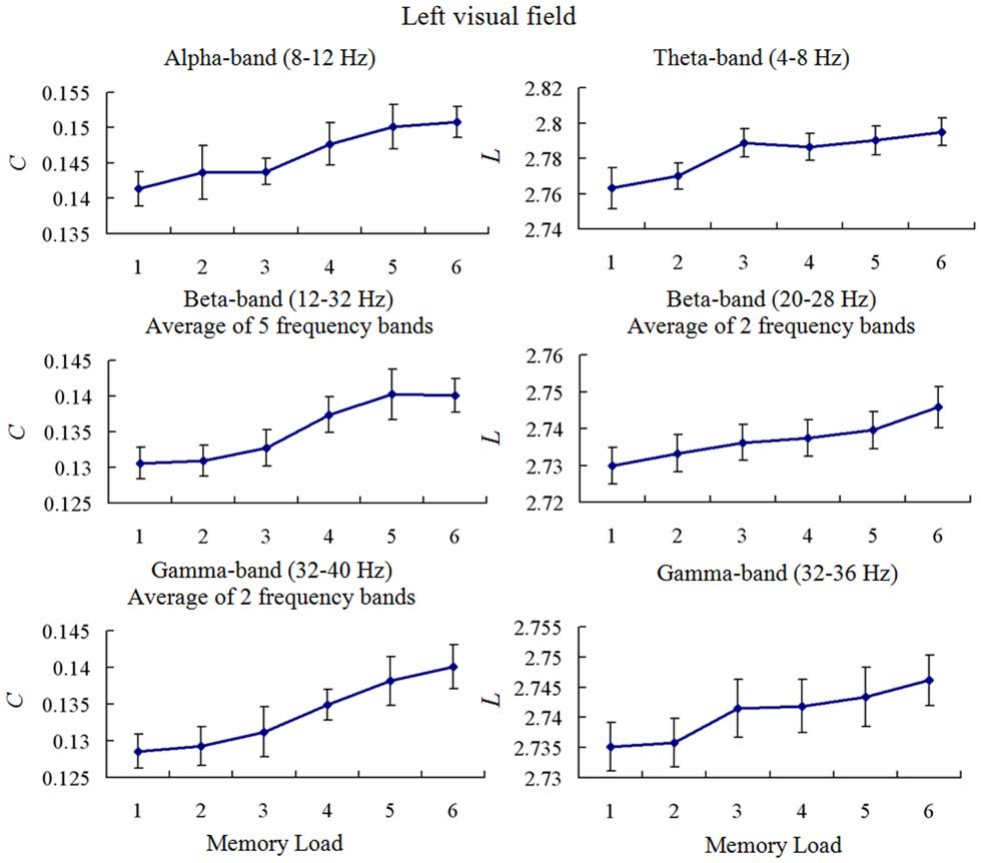
Mean clustering coefficient *C* and shortest path length *L* in LVF condition. Mean *C* (*left column*) and *L* (*right column*) as a function of memory load during the retention period was shown for significant memory load-dependent frequency bands. The results of Kendall's coefficient of concordance (*W*) and Pearson correlation test indicated that increasing memory load increased mean *C* in alpha, beta, and gamma bands, and increased mean *L* in theta, beta and gamma bands.

For the LVF condition, the results of Kendall's *W* and Pearson correlation test of mean *C* showed that *C* was memory load-dependent, and increasing memory load increased mean *C* in all frequency bands except theta-band (for 8–40 Hz, each *W*>0.272, p<0.003, and positive correlation of each frequency band is significant at the 0.01 level (2-tailed)). The results of Kendall's *W* and Pearson correlation test of mean *L* showed that *L* was memory load-dependent, and increasing memory load increased mean *L* in theta-, beta-, and gamma- bands (for 4–8 Hz, *W* = 0.241, p<0.005; for 20–24 Hz, *W* = 0.382, p<0.0001; for 24–28 Hz, *W* = 0.326, p = 0.0004; for 32–36 Hz, *W* = 0.296, p<0.001; and positive correlation of those frequency bands is significant at the 0.05 level (2-tailed)). Figure 6 illustrates the mean *C* and *L* as a function of memory load for significantly frequency bands. The results of Kendall's coefficient of concordance (*W*) test and Pearson correlation indicated that increasing memory load increased clustering coefficient *C* in alpha-, beta-, and gamma- frequency bands, and increased shortest path length *L* in the theta-, beta-, and gamma- frequency bands. The maximal correlative coefficient between memory load and the *C* was 0.538 under the gamma-band (36–40 Hz). And the maximal correlative coefficient between memory load and the *L* was 0.320 under the theta-band (4–8 Hz).

**Figure 7:**
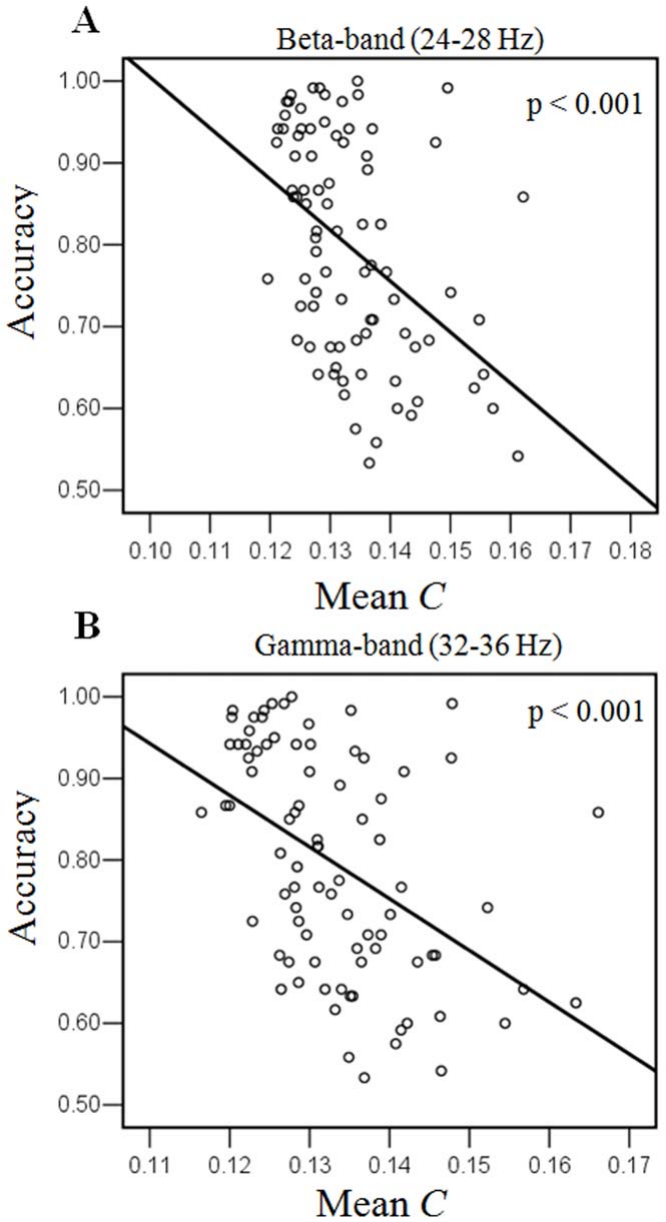
Relationship between the behavioral accuracy and mean *C* in LVF condition. Significantly linear relationship between clustering coefficient *C* and accuracy for (A) beta-band (only band of 24–28 Hz was shown) and (B) gamma-band were illustrated. Each single subject in each memory load condition is marked as a circle, totaling 84 circles. These regressions were significant (p<0.001, Bonferroni corrected), indicating a negative linear correlation between *C* and accuracy.

Multi-linear regressions of the *C/L* and memory load with the behavioral results (RT and accuracy) for LVF condition were performed in all significant frequency bands, respectively. We found that for the *C* there was a significant multi-linear regression between behavioral accuracy and two independents (*C* and memory load) (p<0.05) in beta- and gamma- frequency bands (12–36 Hz). Partial regression coefficients of *C* (p<0.05) and memory load (p<0.0001) were significant, which were negative correlation with accuracy. These linear regressions were significant between accuracy and *C* (p<0.001, Bonferroni corrected). For *L* there was no significant multi-linear regression between behavioral results and two independents (*L* and memory load). Figure 7 illustrates the significantly linear relationship between the *C* and accuracy for beta-band (only band of 24–28 Hz was shown) and gamma-band in LVF condition. Those results indicated that behavioral result was not only dependent on the memory load but also the mean clustering coefficient *C* of the brain functional networks. Increasing mean *C* decreased accuracy in beta- and gamma- frequency bands for LVF memory retention.

**Figure 8:**
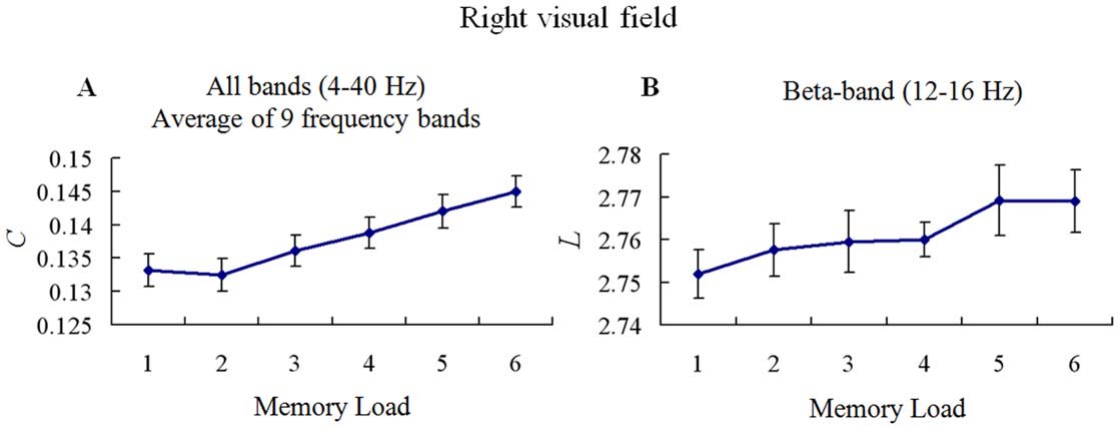
Mean clustering coefficient *C* and shortest path length *L* in RVF condition. Mean *C* (A) and *L* (B) as a function of memory load during the retention period was shown for significant memory load-dependent frequency bands. The results of Kendall's coefficient of concordance (*W*) and Pearson correlation test indicated that increasing memory load increased mean *C* in theta-, alpha-, beta-, and gamma- bands, and increased mean *L* only in beta-band.

For the RVF condition, the results of Kendall's *W* and Pearson correlation test of mean *C* showed that the *C* was memory load-dependent, and increasing memory load increased mean *C* in all frequency bands (for 4–40 Hz, each *W*>0.325, p<0.0004, and positive correlation of each frequency band is significant at the 0.01 level (2-tailed)). The results of Kendall's *W* and Pearson correlation test of mean *L* showed that the *L* was memory load-dependent, and increasing memory load increased mean *L* only in beta-band (for 12–16 Hz, *W* = 0.206, p = 0.013; and positive correlation of this frequency band is significant at the 0.05 level (2-tailed)). Figure 8 illustrates the mean *C* and *L* as a function of memory load for significantly frequency bands. The results of Kendall's coefficient of concordance (*W*) test and Pearson correlation indicated that increasing memory load increased clustering coefficient *C* in theta-, alpha-, beta-, and gamma- frequency bands, and increased shortest path length *L* only in beta frequency band. The maximal correlative coefficient between memory load and *C* was 0.521 under the alpha-band (8–12 Hz). And the maximal correlative coefficient between memory load and the *L* was 0.240 under the beta-band (12–16 Hz).

**Figure 9:**
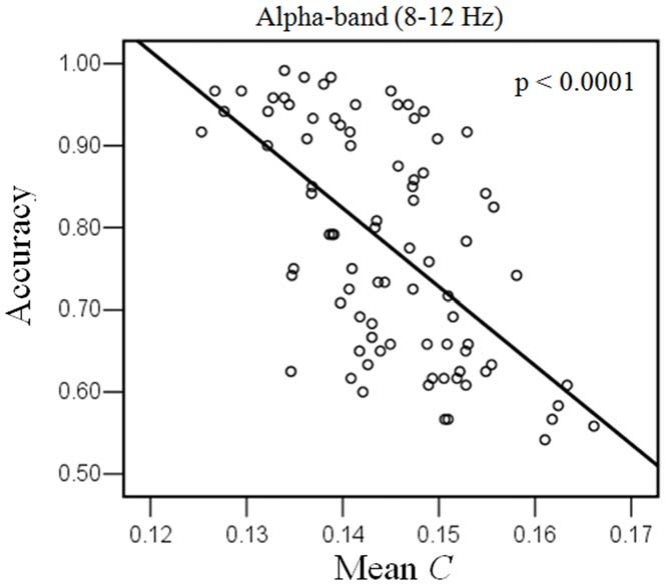
Relationship between the behavioral accuracy and mean clustering coefficient *C* in RVF condition. Significantly linear relationship between mean *C* and accuracy for alpha-band was illustrated. Each single subject in each memory load condition is marked as a circle, totaling 84 circles. This regression was significant (p<0.0001, Bonferroni corrected), indicating a negative linear correlation between *C* and accuracy.

Multi-linear regressions of the *C/L* and memory load with the behavioral results (RT and accuracy) for RVF condition were performed in all significant frequency bands, respectively. We found that for the *C* there was a significant multi-linear regression between behavioral accuracy and two independents (*C* and memory load) (p<0.05) in alpha frequency band (8–12 Hz). Partial regression coefficients of the *C* (p = 0.0003) and memory load (p<0.0001) were significant, which were negative correlation with accuracy. This linear regression were significant between accuracy and *C* (p<0.0001, Bonferroni corrected). For the *L* there was no significant multi-linear regression between behavioral results and two independents (*L* and memory load). Figure 9 illustrates the significantly linear relationship between the *C* and accuracy for alpha-band in RVF condition. Those results indicated that behavioral result was not only dependent on the memory load but also the mean clustering coefficient *C* of the brain functional networks. Increasing mean *C* decreased accuracy in alpha frequency band for RVF memory retention.

### Small-world brain network results

**Figure 10:**
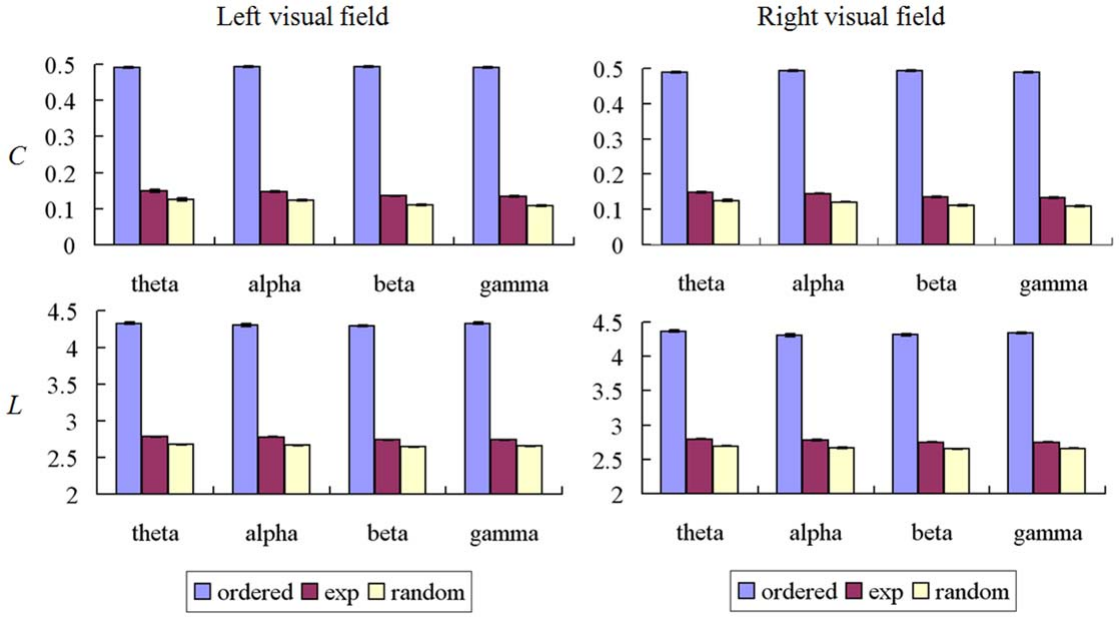
Mean *C* and *L* in ordered, experimental, and random networks. Figure shows the **c**omparison of the experimental clustering coefficient *C* and path length *L* with those of the corresponding values in random and ordered networks for left and right visual field memory conditions. Both *C* and *L* are intermediate between ordered and random networks for all frequency bands, whereas *C* of the EEG is significantly smaller than *C* of ordered networks and significant larger than *C* of random networks, and *L* of the EEG is significantly lower than *L* of ordered networks and larger than *L* of random networks (paired *t*-test, p<0.001, Bonferroni corrected).

The mean clustering coefficient *C* and path length *L* of brain networks of the average load condition in theta-, alpha-, beta- and gamma- frequency bands for two hemi-field conditions, and corresponding *C_ordered_*, *C_random_*, *L_ordered_*, and *L_random_* are shown in the Figure 10. Both the *C* and *L* of brain networks for two visual-fields were intermediate between those of ordered and random networks for four types of frequency bands. The *C* or *L* in left and right visual field conditions had similar pattern compared to those of the random and ordered networks. The *C* of the brain network was significantly smaller than *C_ordered_* and significantly larger than *C_random_*, and the *L* of the brain network was significantly lower than *L_ordered_* and larger than *L_random_* (paired *t*-test, p<0.001, Bonferroni corrected). The results indicated that brain network of those four types of frequency bands had intermediate clustering coefficient *C* and path length *L* compared to ordered and random networks, characteristic of small-world networks [37].

## Discussion

The main goal of this study was to find VWM load-related changes in neural activity and functional connectivity in both hemi-field WM tasks during retention interval by traditional ERP methods and modern graph theoretical techniques. The behavioral data (Figure 2) showed that subjects had more rapid and accurate responses to the low memory load condition than those of the high memory load, indicating that the high memory load task was sufficiently difficult to demand more cognitive resource and effort. Specially, subjects had more rapid and accurate responses to the LVF memory condition, indicating a hemi-field asymmetry in visual object encoding and information maintenance. ERP data provided further evidence that the memory capacity was associated with the neural activity during retention interval over parietal-occipital areas (Figure S1, S2 and Figure 3) [2], [4]. ERP results also showed that memory retention initially began as contralateral predominance, but tended to decrease this effect after a period of time. The results of graph theory measures showed that brain network was not only VWM load-dependent, but also visual-field dependent (Figure 4, 5, 6, 7, 8, 9, 10, and Table 1).

### Memory capacity and ERP amplitude

Previous studies have reported that VWM reaches capacity limit at about 3 colors, 2 shapes, and 2 compound objects, which means that the memory capacity decreases with the increase of object complexity [41]–[43]. In our VWM task, mean memory capacity was about 2 objects, which was estimated by the Cowan's formula or directly by the observation of behavioral accuracy (Figure 2B, accuracy decreased rapidly starting from memory load 2 condition).

Figures S1, S2, and Figure 3 indicated that VWM load-related changes resulted in an alteration of neural activity in the retention interval at parietal-occipital areas. The parietal-occipital areas have been frequently reported to play a role in active maintenance in VWM tasks [44]–[45], in which the superior intraparietal sulcus and the lateral occipital complex are important for encoding and maintaining a variable subset of the attended objects [3]. The mean amplitude difference in ERPs between the ipsilateral and contralateral parietal-occipital electrodes during the retention interval increased with the increase of the memory load and reached a plateau at memory load 2 condition, which equaled to the mean memory capacity in the present task. The above ERP results provided further evidence that there was a direct relationship between memory capacity and neural activity, suggesting that mean amplitude of ERP difference could be a predictor of the memory capacity [4]–[5].

### Changes in brain network density for hemi-field memory retention

The mean degree *K* of brain network was memory load-dependent in beta- and gamma- frequency bands for LVF WM, but in theta-, alpha-, beta-, and gamma- frequency bands for RVF WM (Figure 4). On the one hand, this meant that the inter-region phase synchrony was memory load-dependent and strengthened with increasing memory load in theta-, alpha-, beta-, and gamma- frequency bands [5], [10]–[12]. On the other hand, the results indicated that an increasing VWM load increased the number of significant connections between interconnected brain areas for left and right visual field WM in brain networks in different frequency bands. Specially, increasing memory load in RVF increased network density in theta- and alpha- frequency bands. The current findings demonstrated that phase synchronous networks of theta- and alpha-band were in particular sensitive to the memory load for RVF memory retention.

Working memory requires the simultaneous storage and processing of information, including the central executive functions or attentional control system [1]. Simultaneous EEG-fMRI studies of resting state have found that alpha power is negatively correlated with parietal and frontal cortical activity [46]–[47]. The fronto-parietal network is known to support attentional processes [48]. The functions of alpha rhythms as well as theta rhythms have also been explored in different cognitive tasks, suggesting that the alpha- and theta-band synchrony plays an active role in the attentional-control system [49]–[54]. Furthermore, we found that increasing network density in theta-band for RVF WM was significantly linked to the delay of behavior reaction time (Figure 5). Combined with the behavior results, the decrease of the behavior performance for RVF WM could be the result of the involvement of theta-band phase synchronous network during the information retention period.

### Changes in hubs for hemi-field memory retention

The hubs of left or right visual field memory retention well described the properties of phase synchrony networks, reporting a high density of strong functional core areas. Palva and colleagues have suggested that inter-area phase synchrony in the alpha-, beta-, and gamma-frequency bands among frontoparietal and visual regions could be a systems level mechanism for the information maintenance in VWM for a whole visual field task [5]. They found that major hubs were located to the frontal cortex for alpha-band, right extrastriate regions and left intrparietal sulcus for beta-band, bilateral intraparietal sulcus and left superior parietal gyrus for gamma-band. According to our data (Table 1), despite the low spatial resolution, the similar hubs were found, and the changes in hubs between right and left visual-field retention were found also. In this study, the network hubs in these frequency bands were largely located in frontal, parietal, and visual regions that have been observed in previously study [55]. From Table 1, we found that bilateral frontal and occipital areas were very important for two hemi-fields memory retention, but left parietal areas especially played a more important role in RVF memory retention for alpha-, beta-, and gamma- bands. The enhanced involvement of the left parietal could suggest a compensation mechanism for memory retention to RVF information, though we have not yet solid evidence to demonstrate this hypothesis.

### Changes in graph theory measures for hemi-field memory retention

The clustering coefficient *C* and shortest path length *L* of the brain network under the same network density was memory load-dependent and hemi-field dependent (Figure 6 and Figure 8). The *C* showed significantly lower values in the low memory load condition compared with higher memory load conditions for alpha-, beta-, and gamma- bands for LVF memory retention, but increasing memory load increased *C* in all frequency bands for RVF memory retention. This result indicated that memory load changed local connectedness of brain functional network [16]–[18]. The current findings demonstrated that topological property of theta-band phase synchronous network was in particular sensitive to memory load for RVF memory retention, which was in line with the results of network density. Furthermore, we found that increasing *C* was significantly linked to the decrease of behavior accuracy in beta- and gamma- frequency bands for LVF condition, but in alpha frequency band for RVF condition (Figure 7 and Figure 9). This finding implied that the decrease of behavior accuracy with increasing memory load when subjects performed task may be the result of increasing local connectedness in phase synchronous networks in different frequency bands.

The shortest path length *L* of the brain network was memory load-dependent for theta-, beta-, and alpha- frequency bands in LVF condition, but for beta-band in RVF condition (Figure 6 and Figure 8). Increasing memory load increased *L* in these conditions, which suggested that higher shortest path length might bring the delay of reaction time.

Comparisons of the *C* and *L* of the brain networks with the corresponding values of *C_ordered_*, *C_random_*, *L_ordered_*, and *L_random_* showed that both the *C* and *L* of the brain networks were intermediate between those of ordered and random networks for all frequency bands, characteristic of small-world networks [37]. And there was no difference between left and right visual field conditions (Figure 10). Previous studies in neuroscience based on graph theoretical analysis have reported that anatomical and functional brain networks have the characteristic small-world properties [16]–[18], [37]–[38], [56]–[59]. Small-world neural networks have the potential to facilitate synchronization between distant brain areas and efficient information processing [46], [60]–[61]. Hence, topological properties (*C* and *L*) during the retention interval were load-dependent and hemi-field dependent, and modulated by using diverse modes for different frequency bands.

### The main differences between RVF and LVF working memory tasks

Behavior performance in LVF memory task was better than that of RVF memory task. The reason may come from any process of VWM, including attended direction, information encoding, information retention, and information retrieve. In the present study, we explored the contribution of hemi-field WM in retention period. Interestingly, the load-dependent synchronized theta- and alpha- bands networks were only appeared in RVF condition. This suggests that beta- and gamma- bands interactions of brain network were involved in two visual-field conditions for the control of top-down attention that holds visual information in VWM [9]. In addition, theta- and alpha- bands interactions of the whole brain networks might be especially involved in RVF condition for the extra control of top-down attention, which pointed to the possibility that theta- and alpha- bands synchronization could recruit additional processing resources for information retention of RVF [23], [51]–[52]. Furthermore, the load-dependent synchronized theta-band network density was positive correlation with reaction time only for RVF condition, which provided further evidence that more complex control of top-down attention were required in this condition to obtain additional processing resources, resulting in the delay of reaction time. On the other hand, topological property of theta-band phase synchronous network was in particular sensitive to the memory load for RVF memory retention, which supported the view of important role of theta-band coupling for RVF condition. The load-dependent topological property of alpha-band network was negative correlation with accuracy only for RVF condition, which provided further evidence that alpha-band coupling was also important in this visual field condition, increasing *C* resulting in the decease of behavioral accuracy. This result implied that the topological property (*C*) of alpha-band coupling network could be used as a predictor for the behavioral accuracy for RVF memory task.

### Conclusion

We used traditional ERP and modern graph theoretical technique to distinguish VWM load-related changes in neural activity and functional connectivity of hemi-field VWM during the retention interval. Subjects had more rapid and accurate responses to the LVF memory condition. ERP data provided further evidence that the neural activity in the retention interval over parietal-occipital areas was not only associated with the memory load, but also could be used to predict memory capacity. The results of functional connectivity and graph theory measures showed that brain network of theta-, alpha-, beta-, and gamma- frequency bands was VWM load-dependent and visual-field dependent. The theta- and alpha- bands phase synchrony networks were most predominant in information retention of RVF. Furthermore, only for RVF condition, theta-band brain network density during the retention interval were linked to the delay of reaction time, and the load-dependent topological property of alpha-band network was negative correlation with accuracy, suggesting that synchronous of theta- and alpha- frequency bands may be a strategy of recruitment additional processing resources for information retention of RVF.

Taken together, the results show that the neural activity and phase synchrony measures of functional brain connectivity in theta-, alpha-, beta-, and gamma- frequency bands are not only memory load-dependent, but also dependent on the direction of visual-field memory during human VWM maintenance. And the different frequency bands coupling in the RVF condition during retention period are critical to the decline of behavioral performance. We suggest that the differences in theta- and alpha- bands between LVF and RVF conditions in functional connectivity and topological properties during retention period may result in the decline of behavioral performance in RVF task.

## Supporting Information

Figure S1 **Grand averaged ERP waveforms.** Grand averaged contralateral and ipsilateral ERP waveforms for load 3 at PO7/8 electrodes sites. Negative voltage is plotted upwards. A large negative-going voltage was found in contralateral electrodes to the memorized hemi-field within the time periods for the memory array and retention interval. The two grey rectangles reflect the time periods for the memory and test arrays, respectively.
(TIF)

Figure S2 **ERP difference waves.** ERP difference waves at PO7/8 electrodes for load 1, 2, 3, and 5 (from lowermost line to topmost line). The grey rectangle reflects the measurement window of 300–1000 ms after the onset of the memory array to estimate the mean amplitude of ERP difference for memory load 1 to 6 in the retention interval.
(TIF)

Figure S3 **Map of 128 scalp electrode locations and regions of interest.** ROI was circled by a line. There were eight main brain regions, including left/right frontal, temporal, parietal and occipital.
(TIF)

Figure S4 **Functional connectivity in four frequency bands for two visual-fields.** The mean degree *K* of left visual field condition was 6.47, 12.30, 1.72, and 4.69 for theta-, alpha-, beta-, and gamma- bands, respectively. The mean degree *K* of right visual field condition was 4.48, 10.30, 1.47, and 4.06 for theta-, alpha-, beta-, and gamma- bands, respectively. The networks of left visual field memory had larger connective density than those of right visual field condition.
(TIF)
