# Supplementary figures and images for: Visual Working Memory Load-Related Changes in Neural Activity and Functional Connectivity
Source: PLoS One. 2011 Jul 18;6(7):e22357. doi: 10.1371/journal.pone.0022357 (PMC3138779; doi:10.1371/journal.pone.0022357)

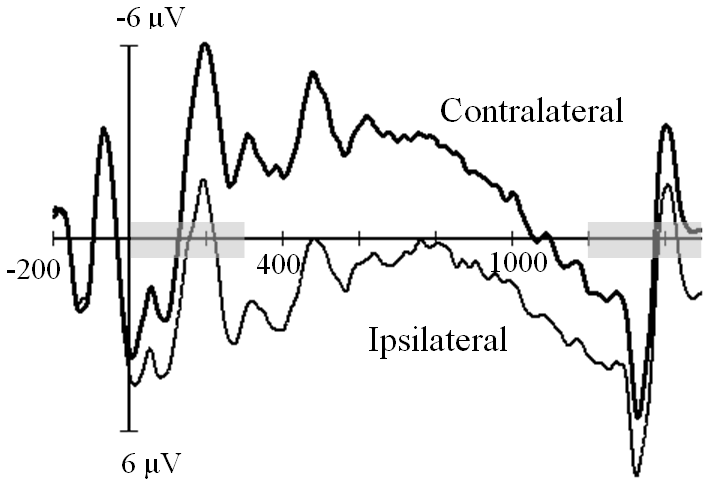

Supplement: Figure S1 — Grand averaged ERP waveforms. Grand averaged contralateral and ipsilateral ERP waveforms for load 3 at PO7/8 electrodes sites. Negative voltage is plotted upwards. A large negative-going voltage was found in contralateral electrodes to the memorized hemi-field within the time periods for the memory array and retention interval. The two grey rectangles reflect the time periods for the memory and test arrays, respectively. (TIF) [file pone.0022357.s001.tif]

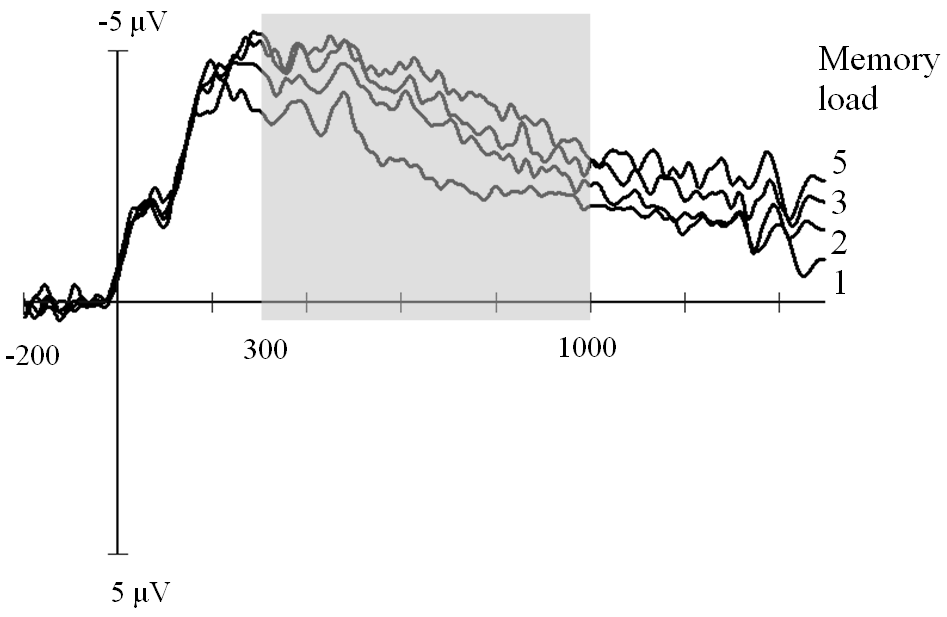

Supplement: Figure S2 — ERP difference waves. ERP difference waves at PO7/8 electrodes for load 1, 2, 3, and 5 (from lowermost line to topmost line). The grey rectangle reflects the measurement window of 300–1000 ms after the onset of the memory array to estimate the mean amplitude of ERP difference for memory load 1 to 6 in the retention interval. (TIF) [file pone.0022357.s002.tif]

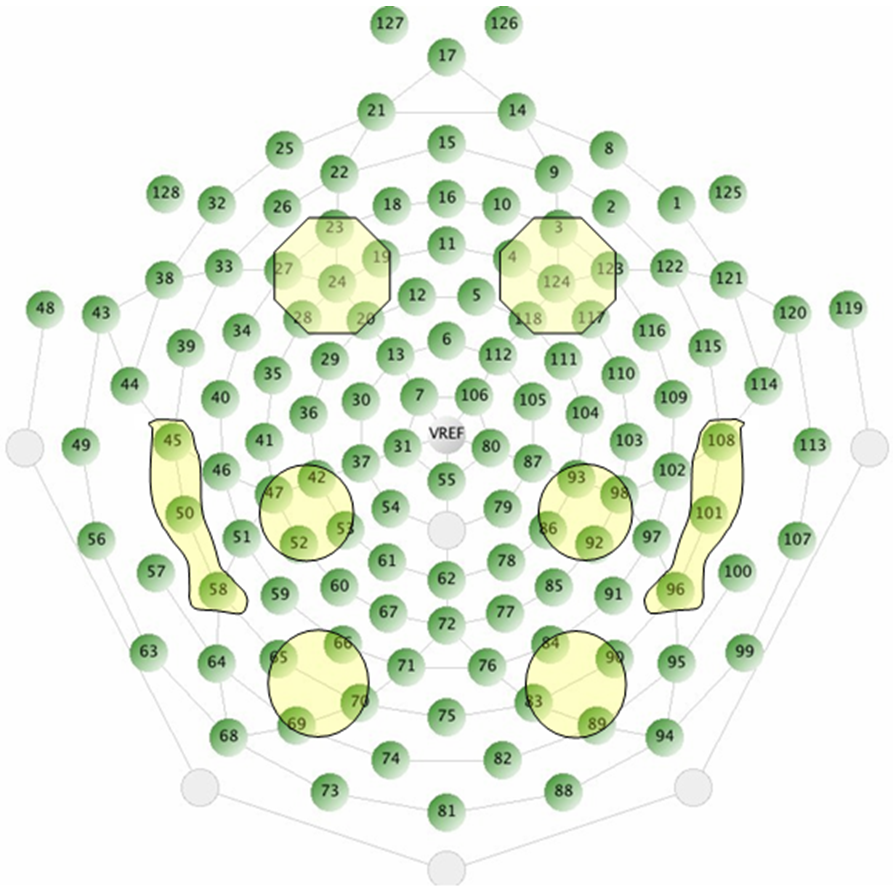

Supplement: Figure S3 — Map of 128 scalp electrode locations and regions of interest. ROI was circled by a line. There were eight main brain regions, including left/right frontal, temporal, parietal and occipital. (TIF) [file pone.0022357.s003.tif]

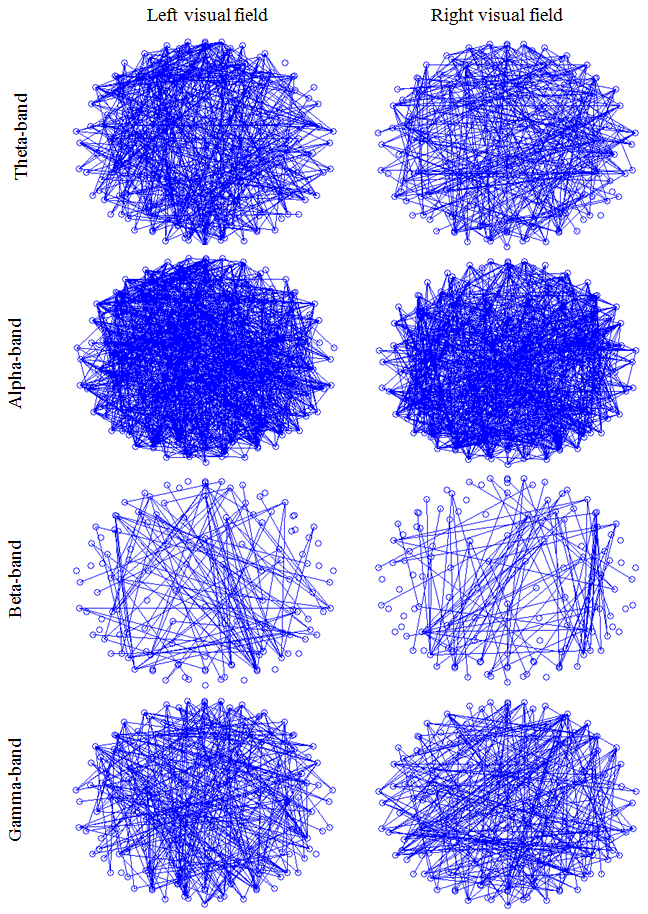

Supplement: Figure S4 — Functional connectivity in four frequency bands for two visual-fields. The mean degree K of left visual field condition was 6.47, 12.30, 1.72, and 4.69 for theta-, alpha-, beta-, and gamma- bands, respectively. The mean degree K of right visual field condition was 4.48, 10.30, 1.47, and 4.06 for theta-, alpha-, beta-, and gamma- bands, respectively. The networks of left visual field memory had larger connective density than those of right visual field condition. (TIF) [file pone.0022357.s004.tif]
